# Comparative Analysis of *FUT1* and *FUT2* Haplotype Diversity in Multi‐Ethnic Populations Via Long‐Read Sequencing

**DOI:** 10.1155/humu/5597086

**Published:** 2025-11-22

**Authors:** Shuang Liang, Weiyi Fu, Fan Wu, Tong Liu, Liyan Sun, Jin Qiu, Yuan Yao, Runjun He, Zhihui Deng, Yanli Ji

**Affiliations:** ^1^ Institute of Transfusion Medicine, Shenzhen Blood Center, Shenzhen, China, itxm.org; ^2^ DAFEI Biotechnology Co., Ltd, Guangzhou, China; ^3^ People’s Hospital of Xinjiang Uygur Autonomous Region, Urumchi, China, xjrmyy.com; ^4^ Institute of Clinical Blood Transfusion, Guangzhou Blood Center, Guangzhou, China

**Keywords:** ethnic diversity, *FUT1*, *FUT2*, genetic variation, haplotype, long-read sequencing

## Abstract

**Background:**

The antigen of H blood group system, synthesized by the fucosyltransferase encoded by *FUT1* and *FUT2* genes, is crucial in transfusion medicine. Traditional sequencing struggles with haplotype determination, necessitating advanced technologies like long‐read sequencing.

**Methods:**

We employed long‐read sequencing to analyze *FUT1* and *FUT2* haplotypes in 154 individuals, including 138 multi‐ethnic donors (46 Chinese Han, 49 Uyghur, and 43 Indian) and 16 para‐Bombay cases.

**Results:**

We successfully reconstructed 8.5 kb *FUT1* and 10.5 kb *FUT2* haplotypes, identifying 44 and 82 single‐nucleotide variants (SNVs), respectively. These SNVs allowed for the classification into four *FUT1* and three *FUT2* SNV patterns, reflecting ethnic diversity. Notably, pattern C for both *FUT1* and *FUT2* showed strong linkage disequilibrium (*R* ^2^ = 0.84), consistent with their close genomic proximity, and was not observed in the Han populations. The prevalence of H‐negative phenotype‐related *FUT2* alleles was markedly lower in Han (1.09%) compared to Uyghur (26.53%) and Indian (50.00%), explaining the rarity of the Bombay phenotype in Han. Furthermore, we found that a single allele named by ISBT can encompass multiple distinct haplotype sequences (spanning various patterns), revealing a level of diversity not captured by traditional SNV‐based classifications.

**Conclusions:**

Our findings highlight the unique genetic patterns within *FUT1/FUT2*, characterized by specific SNVs, suggesting a need for more nuanced approaches to accurately capture genetic variation, evolutionary relationships, and clinical implications. This study establishes long‐read sequencing as pivotal for blood group genetics, revealing population‐specific evolutionary dynamics that clarify ethnic divergence in antigen expression and disease susceptibility.

## 1. Introduction

The H blood group system, governed by *FUT1* and *FUT2* genes, encodes fucosyltransferase responsible for synthesizing H antigens on red blood cells (RBCs) and in secretions, respectively [1]. These antigens serve as precursors for ABO antigens, making their genetic regulation critical for transfusion compatibility and disease association studies [2, 3]. Variations in *FUT1* and *FUT2* can lead to rare phenotypes such as Bombay (absence of H, A, and B antigens) and para‐Bombay (weak/no H antigen on RBCs but preserved secretion status) [4–6]. Over 40 *FUT1* and 20 *FUT2* alleles linked to these phenotypes have been cataloged by the International Society of Blood Transfusion (ISBT), with distinct ethnic prevalence patterns (e.g., Bombay phenotype predominates in Indian populations) [7, 8].

Genotyping for blood group systems has become a standard practice in immunohematology laboratories, offering significant advantages over traditional serological methods [9]. Traditional techniques, such as polymerase chain reaction using sequence specific priming (PCR‐SSP), Bead‐Chip, PCR sequence‐based typing (PCR‐SBT), and next‐generation sequencing (NGS), face limitations in detecting complex structural variations and non‐coding region polymorphisms due to fragmented reads [10–15]. Third‐generation long‐read sequencing (e.g., PacBio SMRT and Oxford Nanopore) overcomes these challenges by generating contiguous reads with an average length of 10–25 kb [16–18]. These advancements enable the identification of structural variations and the creation of comprehensive haplotype sequences. Recent studies have demonstrated its utility in acquiring haplotype and transcriptional sequences such as *ACKR1*, *RHD-RHCE*, and *ABO* genes [19–23] and further resolving complex allelic architectures in the *ABO* and *RHD-RHCE* genes with continuous advance [22, 23]. However, comprehensive haplotype analyses of *FUT1/FUT2* across diverse populations remain limited.

Traditional allele classification and naming schemes, such as those established by the ISBT, are robustly built upon SNVs in coding and splice site regions that directly alter the blood group antigen expression. This focus ensures clinical relevance and utility in transfusion medicine. However, this targeted approach does not encompass the full spectrum of genetic variation, particularly in non‐coding regions or synonymous sites [24]. Consequently, the haplotype diversity and evolutionary history that underpin these clinically defined alleles often remain unexplored. Long‐read sequencing technology now enables the resolution of these complete haplotype sequences, offering a deeper layer of genetic context beyond the core phenotype‐defining variants.

Here, we utilized PacBio long‐read sequencing to comprehensively analyze *FUT1* and *FUT2* haplotypes across a cohort of 154 individuals, encompassing Chinese Han, Uyghur, and Indian donors, and also para‐Bombay individuals. This investigation focused on characterizing the genetic diversity and ethnic‐specific distribution of haplotypes, elucidating the molecular mechanisms driving H‐antigen deficiency phenotypes, and critically assessing the limitations of conventional SNV‐based allele classification systems. The findings demonstrate novel haplotype architectures exhibiting pronounced ethnic stratification and challenge existing nomenclature frameworks, underscoring the necessity of adopting haplotype‐centric methodologies in blood group genomics to advance precision in transfusion medicine and population genetics.

## 2. Materials and Methods

### 2.1. Samples

Peripheral blood samples were collected from 138 blood donors (46 Chinese Han, 49 Uyghur, 43 Indian) and 16 para‐Bombay individuals of Han descent at Shenzhen Blood Center, China. This study was reviewed and approved by the Shenzhen Blood Center Ethics Committee, with the approval number SZBCMEC‐2022‐013. Written informed consent was obtained from all participants, and this method of obtaining informed consent was approved by the Ethical Scientific Committee. All procedures were conducted in accordance with the Declaration of Helsinki. EDTA anticoagulated blood (5 mL) was collected for serologic typing and molecular analyses.

### 2.2. Serologic Testing and DNA Preparation

The routine serologic blood group testing, i.e., red blood cell antigen antibody agglutination assay, is performed to assess the expression strength of the H antigen in all samples utilizing anti‐H monoclonal antibody (Shanghai Hemo‐pharmaceutical Biological Company, Shanghai, China). The forward and reverse ABO blood group typing, utilizing monoclonal anti‐A, anti‐B antibodies, and A, B, and O phenotype cells (Shanghai Hemo‐pharmaceutical & Biological, Shanghai, China), was carried out following standard procedures in 16 para‐Bombay samples.

The genomic DNA was extracted from peripheral blood using the MagNA Pure LC DNA Isolation Kit I (Roche, Basel, Switzerland). Then, DNAs were prepared at a final concentration of 30 ng/*μ*L and stored in Tris‐EDTA buffer for subsequent experiments.

### 2.3. Long‐Range PCR (LR‐PCR) Amplification of *FUT1/FUT2* Genes

Primers spanning the entire *FUT1* (8.5 kb, NG_007510.1) and *FUT2* (10.5 kb, NG_007511.1) genes, including the whole coding region, were designed as follows: *FUT1*‐F: 5 ^′^‐gtccgagtccatcaatggctcgggt‐3 ^′^, *FUT1*‐R: 5 ^′^‐ataatccgctccctcctccgcctcttctg‐3 ^′^, *FUT2*‐F: 5 ^′^‐caacctccgcctcccaagttcaagc‐3 ^′^, and *FUT2*‐R: 5 ^′^‐ctctgcctccctggtgcaaacgattc‐3 ^′^. This primer was selected from three different primer pairs designed for each gene to demonstrating the highest amplification efficiency and the least bias in balance based on quantitative performance. LR‐PCR was performed using KOD FX Neo polymerase (TOYOBO, Japan) under the following conditions: initial denaturation at 94°C for 2 min; 30 cycles of 98°C for 10 s and 68°C for 10 min; final extension at 68°C for 10 min. PCR products were purified with 0.6 × Ampure PB beads (Pacific Biosciences, United States).

### 2.4. Library Preparation and Sequencing Using PacBio Platform

Single‐molecule real‐time (SMRT) libraries were constructed via a streamlined one‐step protocol integrating DNA damage repair, end‐repair, and adapter ligation. The reaction mixture (10 *μ*L total volume) contained 120–250 ng PCR product, 5 *μ*mol/L barcoded adapters (Integrated DNA Technologies), 1 × T4 DNA ligase buffer (Enzymatics, United States), 1 mmol/L ATP (NEB, United States), 200 *μ*mol/L dNTP (NEB, United States), 2.5 U T4 polynucleotide kinase (NEB, United States), 0.75 U T4 DNA polymerase (NEB, United States), and 180 U T4 DNA ligase (Enzymatics, United States). Reactions were sequentially incubated at 37°C (20 min), 25°C (15 min), and 65°C (10 min). Unligated products were removed using Exonuclease I/III (Enzymatics, United States), followed by purification with 0.6 × Ampure PB beads. For multiplexed sequencing, pre‐libraries were pooled by mass, purified twice with 0.45 × Ampure PB beads, and complexed with sequencing enzymes/primers (Sequel Binding Kit 2.2, Pacific Biosciences). DNA‐polymerase complexes (150 pM) were sequenced on the PacBio Sequel II platform (20‐h run).

### 2.5. Data Processing and Variant Calling

Primary analysis of the output data was performed using SMRTLink v10.1.0 software (Pacific Biosciences). Raw reads were first demultiplexed, and barcode sequences were automatically processed at the end of the runs. Subreads were then analyzed to generate HiFi reads using the HiFi application. The HiFi reads were clustered using the Pbaa tool within SMRTLink to correct random errors and improve data quality. Filtered HiFi reads were aligned to the human reference genome (GRCh38.p14, NCBI RefSeq assembly: GCF_000001405.40) using pbmm2 to specifically extract the desired *FUT1* or *FUT2* CCS fragments. The target CCS reads were then re‐aligned to the reference genome of the ISBT *FUT* genes using pbmm2.

For the identification of SNVs and small indels, variant calling was performed using DeepVariant v1.6.0 (https://github.com/google/deepvariant). Alignments of variant and wild‐type molecules were visualized, and genotypes were called using Integrative Genomics Viewer (IGV). Based on the allele frequency (AF) parameter in the extracted VCF files, a heterozygosity threshold of 0.2 was applied. Subsequently, variation patterns in homozygous samples were manually reviewed by experienced analysts to ensure accuracy. Quality control was applied to the VCF files, and only variants meeting strict criteria were retained as true variants. Specifically, variants were required to pass the FILTER status, exhibit a genotype quality (GQ) score greater than 20, have sufficient sequencing support with a read depth (DP) exceeding 100, and show an AF above 0.2.

### 2.6. Haplotype Classification and Statistical Analysis

To ensure that our *FUT1/FUT2* amplification system does not lose haplotypes due to amplification imbalance, we examined the read proportions of minor haplotypes. As shown in Figure S1, even the lowest value was ~ 30%, well above the commonly applied 10%–20% threshold for true heterozygosity, demonstrating robust amplification balance and minimizing the risk of haplotype dropout. In addition, all homozygous samples were manually reviewed by bioinformatics experts to confirm the absence of low‐frequency but structurally distinct alleles. Sequence alignments were performed using SnapGene, and ethnic‐specific SNV frequencies were calculated. Haplotype patterns were defined based on co‐segregating SNVs, and linkage disequilibrium (*R* ^2^) was analyzed using PLINK v1.9. Group differences in haplotype pattern frequencies were evaluated using chi‐square tests. Post hoc pairwise comparisons were performed with Bonferroni correction for multiple testing. A significance level of *p* < 0.05 was considered statistically significant. Statistical significance thresholds were defined as follows: ∗*p* < 0.05, ∗∗*p* < 0.01, and ∗∗∗*p* < 0.001.

## 3. Results

### 3.1. Serologic Typing Results

In all 138 donors, the normal H antigen positive expression was detected on their RBCs (Supplemental Data Table S1). Sixteen para‐Bombay cases exhibited an H‐negative phenotype, accompanied by distinct ABO characteristics (Supplemental Data Table S2), underscoring the serological differences between the common and rare variants.

The experimental strategy and key outcomes are graphically summarized in Figure 1.

**Figure 1:**
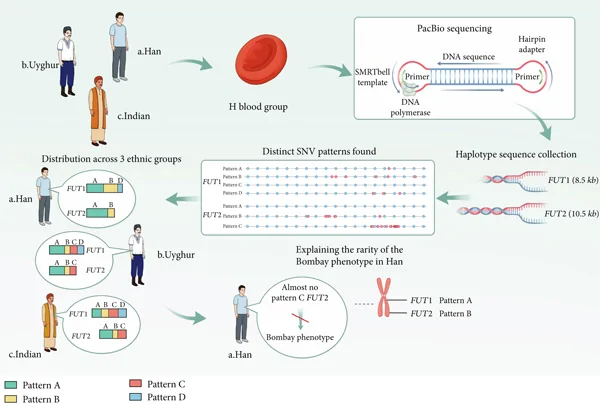
Schematic overview of this study.

### 3.2. Identification of *FUT1/FUT2* Haplotypes

Full‐length *FUT1* (8.5 kb) and *FUT2* (10.5 kb) genes were sequenced via PacBio Sequel II with LR‐PCR amplicons (Figure 2a,b). Sequencing quality metrics (mean PHRED score, subread lengths 5–12 kb) confirmed data reliability (Figure 2c). Reads were mapped to the GRCh38 reference genome and visualized via IGV (Figure 2d–e). Complete haplotype sequences were resolved for all 138 donors (Supplemental Data Table S1) and 16 para‐Bombay individuals (Supplemental Data Table S2). The sequencing quality control data for all samples are provided in Supplemental Data Table S3.

Figure 2Data generation and bioinformatic analysis. (a) Schematic overview of sequencing and haplotype sequences collection of *FUT1/FUT2* (see Methods). (b) Long‐range PCR amplification covering the complete *FUT1/FUT2* gene region. (c) PacBio sequencing data heat map: the horizontal axis represents Polymerase Read Length, the vertical axis represents Longest Subread Length (Log 10), and the color represents the sequencing reads. IGV plots of sequencing results showing variations of (d) *FUT1* and (e) *FUT2* gene.

**Figure:**
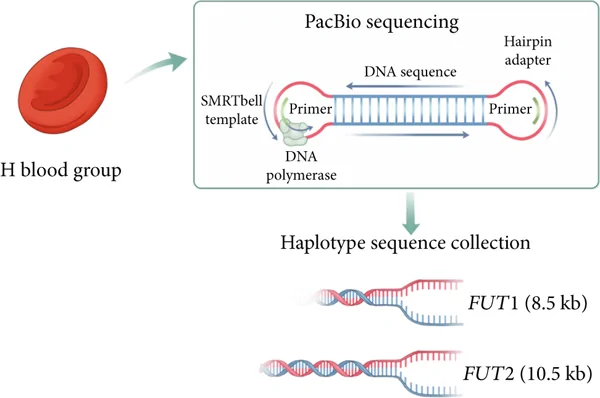
(a)

**Figure:**
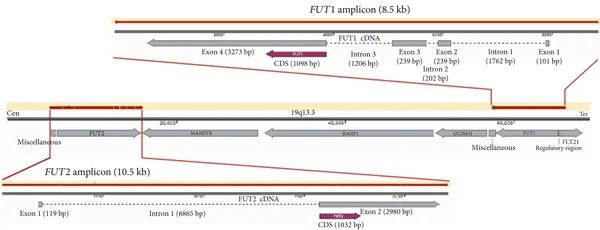
(b)

**Figure:**
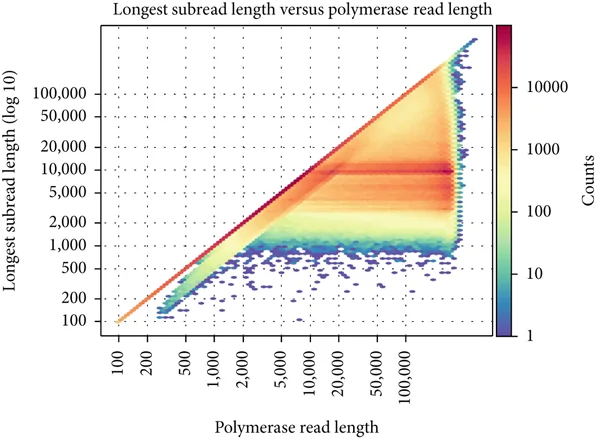
(c)

**Figure:**
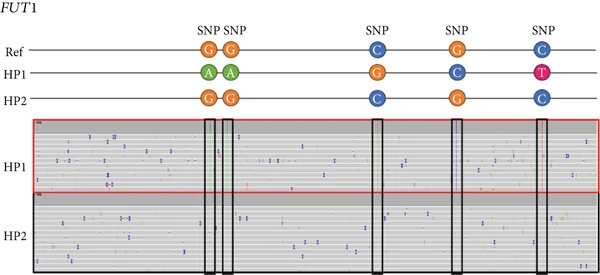
(d)

**Figure:**
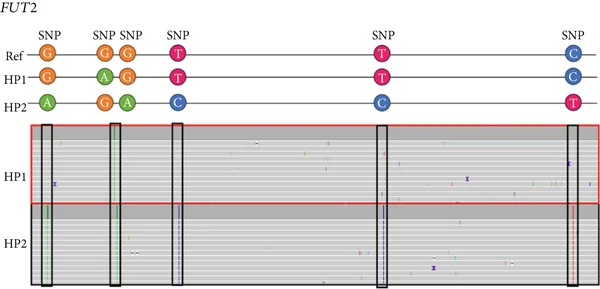
(e)

### 3.3. Genetic Diversity of *FUT1* Haplotypes and the Distribution in the Donors From Three Different Ethnic Groups

Analysis using reference NG_007510.1 identified 44 SNVs in *FUT1* haplotype sequences: 20 in the 5 ^′^‐UTR, 6 in the CDS, and 18 in the 3 ^′^‐UTR (Supplemental Data Table S4), with their frequency distribution among three ethnic groups shown in Figure 3a. Alignment of *FUT1* haplotypes from all samples revealed four distinct SNV patterns labeled Pattern A, B, C, and D (Supplemental Data Table S5), with 10 of the 46 SNVs strongly associated with them. As demonstrated in Figure 3b, Pattern A featured the 3 ^′^‐UTR variant *c.1098+762A>G*; Pattern B combined 5 ^′^‐UTR (*c.1-602C>T*, *c.1-417G>A*, *c.1-35A>T*) and 3 ^′^‐UTR (*c.1098+180*T*>C*, *c.1098+743G>A*, *c.1098+1686C>T*) variants; Pattern D included 3 ^′^‐UTR variants (*c.1098+52G>A*, *c.1098+452G>A*, *c.1098+1204G>A*); Pattern C lacked these linked SNVs, resembling the reference sequence.

Figure 3Genetic diversity and distribution of *FUT1* haplotype sequences across three ethnic groups. NG_007510.1 was used as the reference allele sequence. (a) Frequency of 44 identified SNVs in *FUT1* haplotypes across the three ethnic groups. (b) Schematic representation of four distinct *FUT1* SNV patterns. Red dots highlight distinct SNVs for each pattern. Pattern C represents haplotypes most similar to the reference sequence, lacking distinctive SNVs. (c) Distribution of the four *FUT1* patterns among the three ethnic groups. All SNV information is detailed in Supplemental Data Table S3. Statistical significance of differences in pattern frequencies across ethnic groups was assessed by chi‐square test (*p* < 0.0001). Post hoc pairwise comparisons with Bonferroni correction confirmed significant differences between all ethnic groups (*p* values: Han vs.Uyghur = 0.0003, Han vs.Indian < 0.0001, Uyghur vs.Indian = 0.002).

**Figure:**
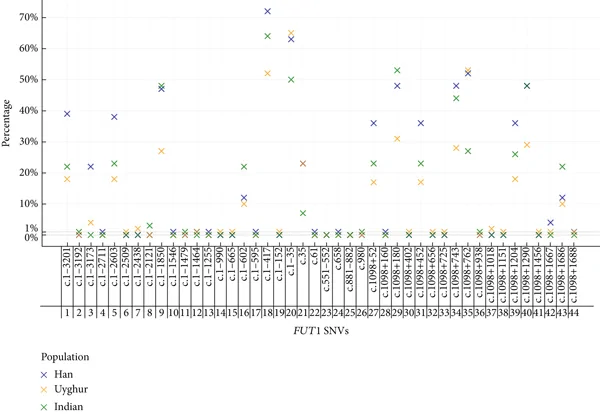
(a)

**Figure:**
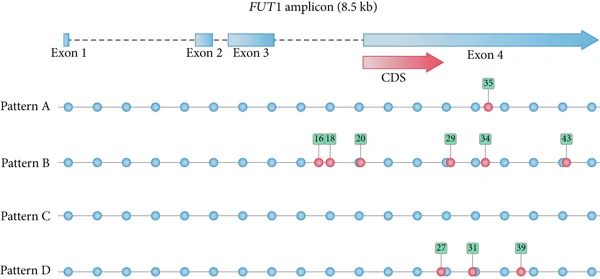
(b)

**Figure:**
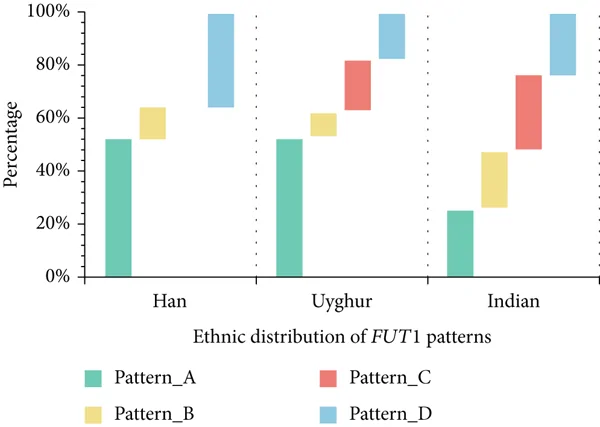
(c)

Among 276 haplotypes (138 samples), 47 distinct *FUT1* haplotype sequences were classified into the four patterns: 16 Pattern A, 12 Pattern B, 4 Pattern C, and 15 Pattern D (Supplemental Data Table S5). Table 1 lists the three most frequent haplotype sequences per pattern (GenBank‐registered). Notably, the pattern distribution varied significantly across ethnic groups (Figure 3c). Pattern A predominated in Han (52.2%) and Uyghur (53.1%) populations but was less frequent in Indians (25.6%). Pattern B showed higher prevalence in Indians (22.1%) versus Han (12.0%) and Uyghur (10.2%). Pattern C was absent in Han but observed in Uyghur (19.4%) and Indian (29.1%) groups. Pattern D was most frequent in Han (35.9%), followed by Indians (23.3%) and Uyghur (17.4%), underscoring population‐specific genetic diversity.

**Table 1:** The most common haplotypes of *FUT1* identified by long‐read sequencing.

**Haplotype SNVs**	**1**	**3**	**5**	**9**	**16**	**18**	**20**	**21**	**23**	**24**	**25**	**26**	**27**	**29**	**31**	**34**	**35**	**39**	**40**	**42**	**43**	**44**	**Random population**			**Para-Bombay (** **n** = ?**)**	**GenBank accession number**
																							**Han (** **n** = ?**)**	**Uyghur (** **n** = ?**)**	**Indian (** **n** = ?**)**		
**NG_007510.1 (*FUT1*)**	**—**	**—**	**—**	**—**	**—**	**—**	**—**	**—**	**—**	**—**	**—**	**—**	**—**	**—**	**—**	**—**	**—**	**—**	**—**	**—**	**—**	**—**					
Pattern A																											
*F1.01.01 (FUT1* ∗ *01)*	—	—	—	—	—	A	T	—	—	—	—	—	—	—	—	—	G	—	—	—	—	—	18	26	15	0	PP894308
*F1.01.02 (FUT1* ∗ *01.02)*	—	—	—	—	—	—	T	T	—	—	—	—	—	—	—	—	G	—	—	—	—	—	20	22	5	0	PP894309
*F1.01.03 (FUT1* ∗ *01)*	—	—	—	—	—	A	T	—	—	—	—	—	—	—	—	—	G	—	—	A	—	—	4	1	0	0	PP894310
Pattern B																											
*F1.02.01 (FUT1* ∗ *01)*	—	—	—	G	T	A	T	—	—	—	—	—	—	C	—	A	—	—	C	—	T	—	4	6	17	0	PP894311
*F1.02.02 (FUT1* ∗ *01)*	—	—	—	—	T	A	T	—	—	—	—	—	—	C	—	A	—	—	C	—	T	—	2	1	0	0	PP898275
*F1.02.03 (FUT1* ∗ *01)*	—	—	—	G	T	A	T	—	—	—	—	—	—	C	—	A	—	—	C	—	T	A	1	0	0	0	PP898276
Pattern C																											
*F1.03.01 (FUT1* ∗ *01)*	—	—	—	—	—	—	—	—	—	—	—	—	—	—	—	—	—	—	—	—	—	—	0	16	21	0	PP907637
*F1.03.02 (FUT1* ∗ *01)*	—	—	—	—	—	—	T	—	—	—	—	—	—	—	—	—	—	—	—	—	—	—	0	2	3	0	PP907638
*F1.03.03 (FUT1* ∗ *01)*	—	—	—	—	—	—	T	T	—	—	—	—	—	—	—	—	—	—	—	—	—	—	0	1	0	0	PP907639
Pattern D																											
*F1.04.01 (FUT1* ∗ *01)*	G	—	C	G	—	A	—	—	—	—	—	—	A	C	A	A	—	A	C	—	—	—	18	3	0	0	PP910828
*F1.04.02 (FUT1* ∗ *01)*	G	T	C	G	—	A	—	—	—	—	—	—	A	C	A	A	—	A	C	—	—	—	7	9	14	0	PP910829
*F1.04.03 (FUT1* ∗ *01)*	G	—	C	G	—	—	—	—	—	—	—	—	A	C	A	A	—	A	C	—	—	—	4	0	1	0	PP937058
*F1.01.15 (FUT1* ∗ *01N.06)*	—	—	—	—	—	A	T	—	delAG	—	—	—	—	—	—	—	G	—	—	A	—	—	0	0	0	16	PP937059
*F1.01.16 (FUT1* ∗ *01N.13)*	—	—	—	—	—	A	T	—	—	—	delTT	—	—	—	—	—	G	—	—	—	—	—	0	0	0	14	PP937060
*F1.01.17 (FUT1* ∗ *01N.16)*	—	—	—	—	—	—	T	T	—	—	—	C	—	—	—	—	G	—	—	—	—	—	0	0	0	2	PP937061

*Note*: Respective major genotypes are indicated by a “–” in the table.

^a^The numbers represent the positions of SNPs.

^b^For each pattern, only the top three most abundant haplotypes were selected and performed sequence alignment. The full version is detailed in Supplemental Data Table S5.

### 3.4. Genetic Diversity of *FUT2* Haplotypes and the Distribution in the Donors From Three Different Ethnic Groups

Analysis of the *FUT2* locus (reference NG_007511.1) identified 82 SNVs, outlined in Supplemental Data Table S5, with ethnic‐specific prevalence patterns (Figure 4a,b). Three haplotype patterns (A, B, C) were defined through sequence alignment (Supplemental Data Table S7). Among the 82 detected SNVs, 30 were notably associated with these patterns. As demonstrated in Figure 4c, Pattern A matched the reference sequence, while Pattern B harbored 5 ^′^‐UTR variants (*c.1-3765InsGTTTT*, *c.1-2554C>T*, *c.1-2481A>G*, *c.1-2475A>G*, *c.1-2193A>C*, *c.1-2157delA*) and a CDS variant (*c.390C>T*). Pattern C exhibited distinct 5 ^′^‐UTR (*c.1-106C>G*, *c.1-69G>C*, *c.1-42C>T*), CDS (*c.138A>G*, *c.183C>T*, *c.395G>A*, *c.706G>A*, *c.927A>G*), and 3 ^′^‐UTR variants (*c.1032+10A>G*, *c.1032+12*T*>C*, *c1032+309C>T*, *c.1032+406*T*>C*, *c.1032+473*T*>C*, *c.1032+547G>A*, *c.1032+1100delC*, *c.1032+1256A>G*, *c.1032+1298*T*>C*, *c.1032+1319C>T*, *c.1032+1384G>C*, *c.1032+1620G>A*, *c.1032+1733A>G*, *c.1032+1765*T*>C*, *c.1032+1981G>A*) (Figure 4c).

Figure 4Genetic diversity and distribution of *FUT2* haplotype sequences across three ethnic groups. NG_007511.1 was used as the reference allele sequence. (a,b) Frequency of 82 identified SNVs in *FUT2* haplotypes across the three ethnic groups. (c) Schematic representation of three distinct *FUT2* SNV patterns. Red dots highlight distinct SNVs for each pattern. (d) Distribution of the three *FUT2* patterns among the three ethnic groups. All SNV information is detailed in Supplemental Data Table S5. Statistical significance of differences in pattern frequencies across ethnic groups was assessed by chi‐square test (*p* < 0.0001). Post‐hoc pairwise comparisons with Bonferroni correction confirmed significant differences between all ethnic groups (*p* values: Han vs.Uyghur < 0.0001, Han vs.Indian < 0.0001, Uyghur vs.Indian = 0.012).

**Figure:**
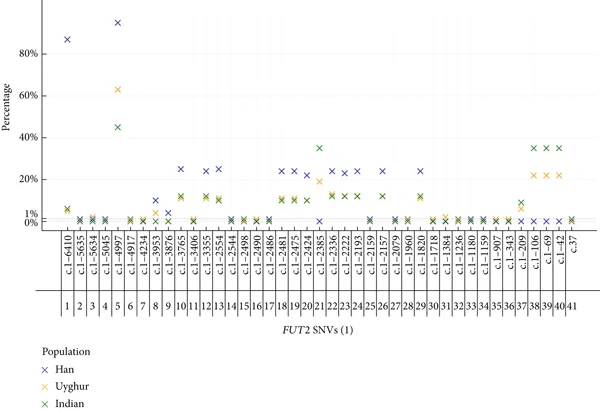
(a)

**Figure:**
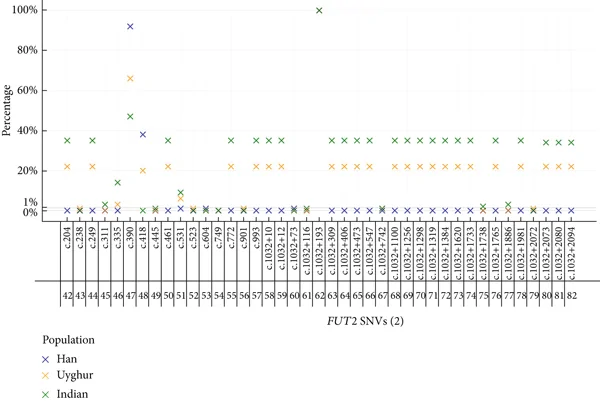
(b)

**Figure:**
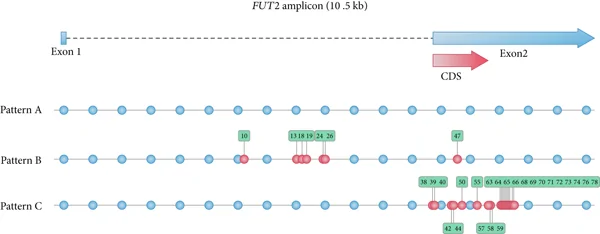
(c)

**Figure:**
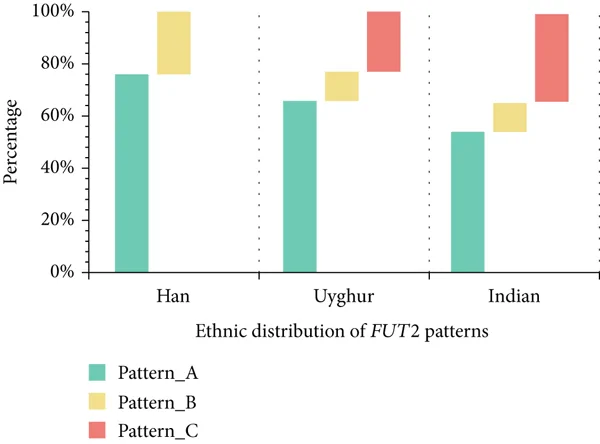
(d)

Among 276 haplotypes (138 samples), 66 unique *FUT2* haplotype sequences were categorized as Pattern A (39), B (18), or C (9) (Supplemental Data Table S7). Table 2 lists the top three haplotype sequences for each pattern, showing how SNVs differ across ethnic groups and relate to H antigen related phenotypes. Ethnic distribution varied significantly (Figure 4d): Pattern A predominated in Han (76.1%), Uyghur (66.3%), and Indian (54.7%) populations. Strikingly, Pattern C was absent in Han but observed in Uyghur (22.5%) and Indian (34.9%) groups.

**Table 2:** Haplotypes of *FUT2* identified by long‐read sequencing.

**No.**	**1**	**2**	**5**	**8**	**9**	**10**	**13**	**18**	**19**	**21**	**24**	**26**	**38**	**39**	**40**	**42**	**44**	**47**	**48**	**50**	**Random population**			**Para-Bombay (** **n** = 32**)**	**GenBank accession number**
**Han (** **n** = 92**)**	**Uyghur (** **n** = 98**)**	**Indian (** **n** = 86**)**																							
**NG_007511.1(*FUT2*)**	**—**	**—**	**—**	**—**	**—**	**—**	**—**	**—**	**—**	**—**	**—**	**—**	**—**	**—**	**—**	**—**	**—**	**—**	**—**	**—**					
Pattern A																									
*F2.01.01 (FUT2* ∗ *01* W*.02.01)*	C	—	T	—	—	—	—	—	—	—	—	—	—	—	—	—	—	T	T	—	32	1	0	0	PP937062
*F2.01.02 (FUT2* ∗ *01)*	C	—	T	—	—	—	—	—	—	—	—	—	—	—	—	—	—	T	—	—	19	1	1	0	PP937063
*F2.01.03 (FUT2* ∗ *01)*	—	—	T	—	—	—	—	—	—	—	—	—	—	—	—	—	—	T	—	—	4	18	10	0	PP937064
Pattern B																									
*F2.02.01 (FUT2* ∗ *01)*	C	—	T	—	—	InsGTTTT	T	G	G	—	C	DelA	—	—	—	—	—	T	—	—	6	0	2	0	PP937065
*F2.02.02 (FUT2* ∗ *01)*	C	—	T	C	—	InsGTTTT	T	G	G	—	C	DelA	—	—	—	—	—	T	—	—	5	1	0	0	PP937066
*F2.02.03 (FUT2* ∗ *01)*	C	—	T	—	T	InsGTTTT	T	G	G	—	C	DelA	—	—	—	—	—	T	—	—	4	0	0	0	PP937068
Pattern C																									
*F2.03.01 (FUT2* ∗ *01N.02)*	—	—	—	—	—	—	—	—	—	T	—	—	G	C	T	G	T	—	—	A	0	15	24	0	PP937069
*F2.03.02 (FUT2* ∗ *01N.02)*	—	—	—	—	—	—	—	—	—	—	—	—	G	C	T	G	T	—	—	A	0	3	0	0	PP937070
*F2.03.03 (FUT2* ∗ *01N.02)*	C	—	—	—	—	—	—	—	—	T	—	—	G	C	T	G	T	—	—	A	0	1	2	0	PP937071
*F2.01.39 (FUT2* ∗ *09)*	C	—	T	—	—	—	—	—	—	—	—	—	—	—	—	—	—	T	—	—	0	0	0	14	PP937072
*F2.02.18 (FUT2* ∗ *01)*	C	A	T	C	—	InsGTTTT	T	G	G	—	C	DelA	—	—	—	—	—	T	—	—	0	0	0	18	PP937073
**No.**	**54**	**55**	**57**	**58**	**59**	**62**	**63**	**64**	**65**	**66**	**68**	**69**	**70**	**71**	**72**	**73**	**74**	**76**	**78**	**/**	**Random population**			**Para-Bombay (** **n** = 32**)**	**GenBank accession number**
**NG_007511.1(*FUT2*)**	**—**	**—**	**—**	**—**	**—**	**—**	**—**	**—**	**—**	**—**	**—**	**—**	**—**	**—**	**—**	**—**	**—**	**—**	**—**	**/**	**Han (** **n** = 92**)**	**Uyghur (** **n** = 98**)**	**Indian (** **n** = 86**)**		
Pattern A																									
*F2.01.01 (FUT2* ∗ *01W.02.01)*	—	—	—	—	—	G	—	—	—	—	—	—	—	—	—	—	—	—	—	/	32	1	0	0	PP937062
*F2.01.02 (FUT2* ∗ *01)*	—	—	—	—	—	G	—	—	—	—	—	—	—	—	—	—	—	—	—	/	19	1	1	0	PP937063
*F2.01.03 (FUT2* ∗ *01)*	—	—	—	—	—	G	—	—	—	—	—	—	—	—	—	—	—	—	—	/	4	18	10	0	PP937064
Pattern B																									
*F2.02.01 (FUT2* ∗ *01)*	—	—	—	—	—	G	—	—	—	—	—	—	—	—	—	—	—	—	—	/	6	0	2	0	PP937065
*F2.02.02 (FUT2* ∗ *01)*	—	—	—	—	—	G	—	—	—	—	—	—	—	—	—	—	—	—	—	/	5	1	0	0	PP937066
*F2.02.03 (FUT2* ∗ *01)*	—	—	—	—	—	G	—	—	—	—	—	—	—	—	—	—	—	—	—	/	4	0	0	0	PP937068
Pattern C																									
*F2.03.01 (FUT2* ∗ *01N.02)*	—	A	G	G	C	G	T	C	C	A	delC	G	C	T	C	A	G	C	A	/	0	15	24	0	PP937069
*F2.03.02 (FUT2* ∗ *01N.02)*	—	A	G	G	C	G	T	C	C	A	delC	G	C	T	C	A	G	C	A	/	0	3	0	0	PP937070
*F2.03.03 (FUT2* ∗ *01N.02)*	—	A	G	G	C	G	T	C	C	A	delC	G	C	T	C	A	G	C	A	/	0	1	2	0	PP937071
*F2.01.39 (FUT2* ∗ *09)*	A	—	—	—	—	G	—	—	—	—	—	—	—	—	—	—	—	—	—	/	0	0	0	14	PP937072
*F2.02.18 (FUT2* ∗ *01)*	—	—	—	—	—	G	—	—	—	—	—	—	—	—	—	—	—	—	—	/	0	0	0	18	PP937073

*Note*: Respective major genotypes are indicated by a “–” in the table.

^a^The numbers represent the positions of SNPs.

^b^For each pattern, only the top three most abundant haplotypes were selected and performed sequence alignment. The full version is detailed in Supplemental Data Table S7.

To better understand the relationship and potential co‐occurrence of these patterns within the same ethnic groups, we further investigated the linkage between *FUT1* and *FUT2*. Linkage analysis revealed strong disequilibrium (*R* ^2^ = 0.84) between *FUT1* Pattern C and *FUT2* Pattern C (Figure 5), which may explain their co‐absence in the Chinese Han population.

**Figure 5:**
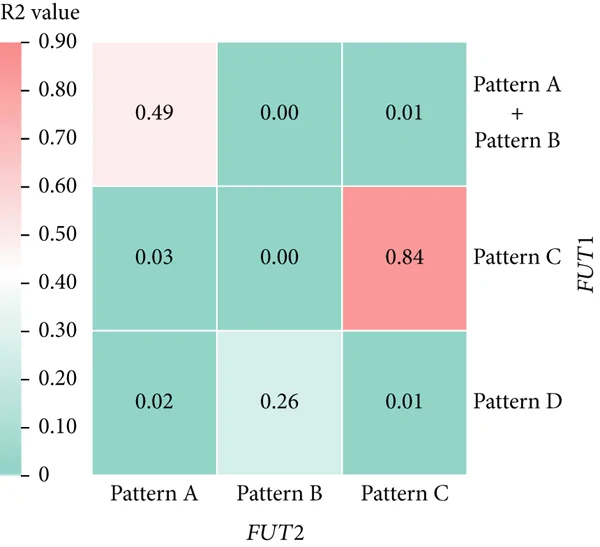
Heatmap of linkage relationship between SNV patterns of *FUT1* and *FUT2*.

### 3.5. *FUT2* Null Alleles and Its Diversity for Phenotypic Implications

Analysis of *FUT2* haplotypes across three different ethnic groups (Table 3) revealed striking disparities in H‐negative allele frequencies: Han (1.09%), Uyghur (26.53%), and Indian (50.00%) populations, which is consistent with the rarity of the Bombay phenotype in Chinese Han population. In Uyghur and Indian cohorts, H‐negative alleles predominantly fell into Pattern C (80.77% and 69.77%, respectively), whereas Han donors exclusively exhibited Patterns A/B, which displayed differences in origin among different ethnic groups.

**Table 3:** Distribution of *FUT2* haplotypes based on the predicted phenotypes.

**Predicted phenotype**	**Allele**	**Nucleotide change in CDS**	**Han (** **N** = 92**)**			**Uyghur (** **N** = 98**)**			**Indian (** **N** = 86**)**		
			**Pattern A (** **n** = 70**)**	**Pattern B (** **n** = 22**)**	**Pattern C (** **n** = 0**)**	**Pattern A (** **n** = 65**)**	**Pattern B (** **n** = 11**)**	**Pattern C (** **n** = 22**)**	**Pattern A (** **n** = 47**)**	**Pattern B (** **n** = 9**)**	**Pattern C (** **n** = 30**)**
H+	*FUT2* ∗ *01*	*—*	7	0	0	8	0	0	4	0	0
	*FUT2* ∗ *01*	*c.390C>T*	26	22	0	28	10	0	18	9	0
	*FUT2* ∗ *02*	*c.37G>A*; *c.390C>T*	0	0	0	0	0	0	1	0	0
	*FUT2* ∗ *NEW*	*c.390C>T*; *c.513C>T*	1	0	0	5	0	0	8	0	0
	Total (*n* and frequency)		56 (60.87%)			51 (52.04%)			40 (46.51%)		

H+w	*FUT2* ∗ *01W.02.01*	*c.390C>T*; *c.418A>T*	35	0	0	20	0	0	0	0	0
	*FUT2* ∗ *01W.01*	*c.311C>T*; *c.390C>T*	0	0	0	0	0	0	3	0	0
	Total (*n* and frequency)		35 (38.04%)			20 (20.41%)			3 (3.49%)		

H‐	*FUT2* ∗ *01N.04*	*c.390C>T*; *c.604C>T*	1	0	0	0	0	0	0	0	0
	*FUT2* ∗ *01N.15*	*c.335C>T*	0	0	0	3	0	0	12	0	0
	*FUT2* ∗ *01N.13*	*c.390C>T*; *c.513C>T*; *c.901G>A*	0	0	0	1	0	0	0	0	0
	*FUT2* ∗ *01N.02*	*c.204A>G*; *c.249C>T*; *c.461G>A*; *c.772G>A*; *c.993A>G*	0	0	0	0	0	21	0	0	30
	*FUT2* ∗ *01N.02*	*c.204A>G*; *c.238G>A*; *c.249C>T*; *c.461G>A*; *c.772G>A*; *c.993A>G*	0	0	0	0	1	0	0	0	0
	*FUT2* ∗ *01N.17*	*c.390C>T*; *c.445G>A*	0	0	0	0	0	0	1	0	0
	Total (*n* and frequency)		1 (1.09%)			26 (26.53%)			43 (50.00%)		

Undefined new allele	*FUT2* ∗ *NEW*	*c.357C>T*; *c.490C>T*	0	0	0	0	1	0	0	0	0
	Total (*n* and frequency)		0			1 (1.02%)			0		

Traditional classification of *FUT2* haplotype sequences (based on coding SNVs and antigen phenotypes) resolved 66 sequences into 13 allele types (as detailed in Table 3). In contrast, *FUT1* displayed limited diversity, with only four alleles (*FUT1* ∗ *01*, *FUT1* ∗ *01.02*, *FUT1* ∗ *NEW*, and *FUT1* ∗ *01*W*.09*) identified among 47 haplotype sequences (Supplemental Data Table S5). Notably, some *FUT2* alleles spanned multiple patterns; for example, the allele *FUT2* ∗ *01*, characterized by the SNV *c.390C>T*, was observed 16 haplotypes in Pattern A and 17 in Pattern B, comprising a total of 33 distinct sequences. These findings underscore the higher evolutionary plasticity of *FUT2* compared to *FUT1*, with population‐specific allele distributions shaping H‐antigen expression variability.

### 3.6. Genetic Diversity Patterns of *FUT1* and *FUT2* Among Chinese Para‐Bombay Individuals

The para‐Bombay phenotype, characterized by silencing *FUT1* but functional *FUT2*, differs genetically from the Bombay phenotype (dual *FUT1/FUT2* silencing). Analysis of 32 haplotypes (16 samples with H‐negative phenotype) revealed that all *FUT1* sequences harbored silencing variants, classified as *FUT1* ∗ *01*N*.06* (*n* = 16), *FUT1* ∗ *01N.13* (*n* = 14), and *FUT1* ∗ *01N.37* (*n* = 2) under ISBT nomenclature (Supplemental Data Table S2). These variants consistently aligned with Pattern A (Figure 6a), characterized by extra specific SNVs summarized in Table 1.

Figure 6Genotype identified in Chinese para‐Bombay individuals. Long‐read sequencing of 16 para‐Bombay samples yielded 32 haplotypes for each of *FUT1* and *FUT2*. Alignment analysis revealed (a) three *FUT1* haplotype sequences and (b) two *FUT2* haplotype sequences. Only the SNVs in the CDS region were marked.

**Figure:**
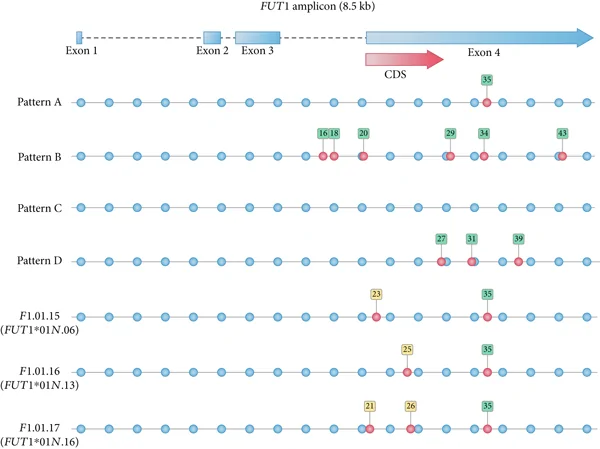
(a)

**Figure:**
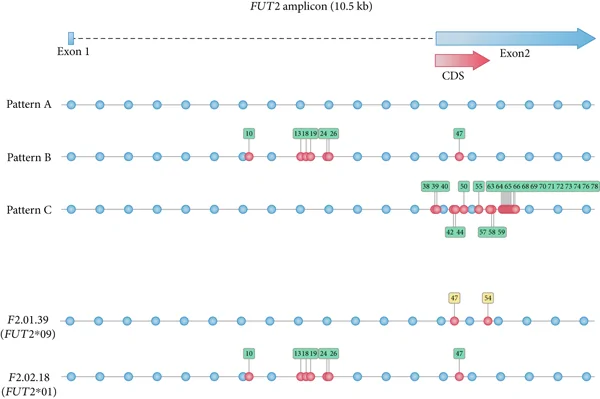
(b)

For *FUT2*, haplotypes segregated into two types: *FUT2* ∗ *01* (*c.390C>T*, *n* = 18) and *FUT2* ∗ *01.09* (*c.390C>T*, *c.749G>A*, *n* = 14) (Supplemental Data Table S7). The *c.390C>T* variant is synonymous, whereas *c.749G>A* (p.Arg239Gln) did not impair H‐antigen expression based on ISBT database. No *FUT2* silencing variants were detected, confirming the para‐Bombay phenotype. Strikingly, these *FUT2* haplotype sequences (classified as Patterns B and A, respectively; Figure 6b) were exclusively identified in para‐Bombay individuals and absent in population‐wide samples. Notably, the *c.749G>A* SNV occurred at a high frequency (43.75%, 14/32) in para‐Bombay cases but was undetected in H‐positive Han donor controls (Figure 6b).

## 4. Discussion

In transfusion medicine, understanding of the genetic basis of blood group antigens is essential for deciphering polymorphisms, ensuring transfusion safety, and exploring disease associations. Traditional genotyping methods such as PCR‐SSP, SBT, and high‐throughput NGS have significantly advanced the identification of genetic variations. However, these methods face challenges in resolving haplotypes, which are critical for understanding the functional interplay of variants and their effect on blood group phenotypes. This limitation stems from their reliance on short‐read sequencing, which complicates phasing of distal variants. In contrast, our study leveraged PacBio long‐read sequencing technology to generate full‐length haplotypes of the *FUT1* and *FUT2* genes, bypassing the need for labor‐intensive cloning or family‐based validation.

We successfully obtained 308 full‐length haplotype sequences for both *FUT1* and *FUT2* from 154 individuals, including 46 Chinese Han, 49 Uyghur, 43 Indian, and 16 Chinese para‐Bombay samples. Using Smrtlink tools for quality control and the IGV for alignment to the GRCh38 reference genome, we achieved high‐resolution visualization of genetic variants. This approach not only confirmed known variants but also revealed novel structural variations and non‐coding SNVs that traditional methods often overlook. For instance, in *FUT1* gene, we identified 46 SNVs distributed across the 5 ^′^‐UTR, coding regions, and 3 ^′^‐UTR, while *FUT2* harbored 83 SNVs with distinct frequencies in different ethnic groups. These findings underscore the utility of long‐read sequencing in capturing the full genetic landscape of blood group systems.

### 4.1. Population‐Specific Haplotype Patterns and Evolutionary Insights

A striking discovery of this study was the ethnic divergence in haplotype patterns. For *FUT1* gene, the haplotypes with four distinct patterns (A–D) were identified: Patterns A and B are more common in the Han and Uyghur populations but less frequent in Indians. Pattern D is prevalent in the Han but occurs at lower frequencies in Uyghurs and Indians. Pattern C is virtually absent in the Han but constitutes a significant proportion of haplotypes in both Uyghur and Indian groups. Similarly, *FUT2* haplotypes clustered into three patterns (A–C), with Pattern C completely absent in Han individuals but accounting for 22.5% and 34.9% of Uyghur and Indian haplotypes, respectively. The observed strong linkage disequilibrium (*R* ^2^ = 0.84) between *FUT1* pattern C and *FUT2* Pattern C haplotypes is consistent with the close genomic distance (~ 40 kb) between the two genes, likely reflecting shared ancestry and limited historical recombination. Given this tight genetic linkage and the high frequency of Pattern C in Indian populations, we hypothesize that these haplotypes may have originated from South Asian ancestors, with subsequent genetic drift or selective pressures leading to their absence in Han Chinese.

### 4.2. The Para‐Bombay Phenotype: Genetic Mechanisms and Diagnostic Implications

The haplotype sequencing of the *FUT1* and *FUT2*, provides insights into the genetic basis underlying H blood group antigen expression. In this study, all H‐negative individuals harbored homozygous or compound heterozygous *FUT1* silencing haplotypes but retained functional *FUT2*, confirming their para‐Bombay status. According to the previous reports, nearly all identified Bombay phenotype cases, carried both *FUT1* and *FUT2* null alleles, are of Indian descent, with rare instances found in the Chinese Han population. This difference is due to distinct genetic patterns: most silenced *FUT2* haplotypes fall into Pattern C, which was absent in our Han samples that only showed Patterns A and B. This explains why the Bombay phenotype is virtually rare‐existent among the Chinese Han population. Notably, the *FUT2* c.749G>A variant (p.Arg239Gln) occurred at 43.75% in Chinese para‐Bombay cases but was absent in Chinese Han controls. Our haplotype analysis revealed that this variant is in strong linkage disequilibrium with the *FUT1* ∗ *01*N*.13* allele on the same chromosomal background. This specific *FUT1* ∗ *01N.13* allele was first described in individuals of Chinese ethnicity in Taiwan [25], confirming its Chinese origin. This explains the high frequency of this haplotype in our Shenzhen‐based cohort and its absence in the general population. This specific association suggests its utility as a diagnostic marker for this para‐Bombay haplotype. These findings underscore the unique genetic makeup associated with distinct phenotypes and highlight the importance of application of long‐read sequencing to obtain whole‐gene haplotypes for a better understanding of blood group diversity and its distribution across various populations.

### 4.3. Haplotype Diversity Beyond the Allele Nomenclature of ISBT

The ISBT blood group allele nomenclature is purposefully designed based on phenotypically relevant variations in the coding and splice site regions, providing a critical framework for transfusion medicine. Our study leverages long‐read sequencing to reveal the extensive haplotype diversity that exists within this nomenclature. For instance, all haplotypes of the *FUT1* ∗ *01* reference allele (defined by the absence of coding mutations) could be classified into four distinct patterns (A‐D) based on their non‐coding SNVs. Similarly, the *FUT2* ∗ *01* allele (which includes haplotypes with the synonymous variation c.390C>T) comprised 33 unique haplotype sequences spanning Patterns A (*n* = 16) and B (*n* = 17). This demonstrates that a single ISBT allele name can encompass a multitude of haplotypes with rich sequence diversity in non‐coding regions.

These patterns of diversity are further shaped by population‐specific evolutionary histories. For example, the alleles *FUT2* ∗ *01*N*.02* (c.204A>G, c.249C>T, c.461G>A, c.772G>A, c.993A>G) and *FUT2* ∗ *01N.04* (c.390C>T, c.604C>T) were prevalent in Uyghur (22.45%) and Indian (34.88%) populations but were absent or very rare in Chinese Han individuals. Our analysis of their full haplotype sequences provides a clearer picture of their distinct evolutionary origins, which is not fully captured by their ISBT definitions alone. These findings underscore the value of haplotype‐resolved sequencing in complementing the existing nomenclature by adding a layer of phylogenetic and population‐genetic context.

### 4.4. Clinical Relevance: Haplotype Context and Disease Associations

Beyond transfusion medicine, *FUT2* polymorphisms have been linked to diseases such as Crohn’s disease, ulcerative colitis (UC), primary sclerosing cholangitis, bacterial adhesion, and plasma levels of vitamin B12 [25–31]. This is supported by both genetic and functional studies. For instance, Hu et al. reported an association between *FUT2* polymorphisms and UC susceptibility in a Southeast Chinese population [33], while Li et al. demonstrated that *FUT2*‐mediated fucosylation is crucial for maintaining intestinal barrier integrity and that its enhancement can protect against inflammation [34]. However, the genetic variant *c.357C>T* was found to be significantly associated with UC in the Han population, while shown no association in the Uyghur population [15]. The specific cause of this population difference remains unknown. In our cohort, the genetic variant *c.357C>T* of *FUT2* is primarily found in Patterns A and B. While the frequencies of Pattern A are similar between the Han and Uyghur populations, Pattern B is much more prevalent in the Han population. Therefore, we speculate that this disparity in Pattern B frequencies may explain the increased association with UC observed in the Chinese Han population compared to the Uyghur population. Therefore, whole‐gene haplotype sequencing provides a comprehensive view of genetic variations and phenotypic outcomes.

## 5. Conclusion

This study demonstrates the utility of PacBio long‐read sequencing in resolving *FUT1/FUT2* haplotype sequences and ethnic‐specific SNV patterns, transcending SNV‐centric limitations. These insights advance precision diagnostics, inform transfusion strategies, and illuminate evolutionary drivers of blood group diversity, underscoring the need for haplotype‐aware approaches in genetic and clinical research.

## Conflicts of Interest

The authors declare no conflicts of interest.

## Author Contributions

Conceptualization: S.L.; methodology: S.L., L.S.; writing—original draft preparation: S.L.; data curation: W.F., Y.Y.; software: W.F., R.H.; validation: W.F., F.W.; visualization: T.L.; investigation: L.S., J.Q.; supervision: Z.D.; writing—reviewing and editing: Y.J. S.L. and W.F. equally contributed to this study.

## Funding

This study is supported by the Basic and Applied Basic Research Foundation of Guangdong Province, 2022A1515110195; Natural Science Foundation of Shenzhen Municipality, JCYJ20230807154000002; Guangdong Medical Foundation (No. B2025582), Shenzhen Key Medical Discipline Construction Fund, SZXK070; and Sanming Project of Medicine in Shenzen Municipality, SZSM202311032.

## Supporting Information

Additional supporting information can be found online in the Supporting Information section.

## Supporting information


**Supporting Information 1** Table S1: Phenotype and genotype identified in random blood donors from three different ethnic groups. Table S2: Phenotype and genotype identified in Chinese para‐Bombay individuals. Table S3: Summary of PacBio sequencing metrics and quality control for the *FUT1* and *FUT2* gene regions across all 138 random collected donors. Table S4: SNVs of *FUT1* identified by long‐read sequencing. Table S5: Haplotype analysis of *FUT1* and its distribution patterns across three ethnic groups. Table S6: SNVs of *FUT2* identified by long‐read sequencing. Table S7: Haplotype analysis of *FUT2* and its distribution patterns across three different ethnic groups.

**Supporting Information 2**  

## Data Availability

The data that support the findings of this study are available from the corresponding author upon reasonable request.
