# Supplementary material for: Comparative Analysis of FUT1 and FUT2 Haplotype Diversity in Multi‐Ethnic Populations Via Long‐Read Sequencing
Source: Hum Mutat. 2025 Nov 22;2025:5597086. doi: 10.1155/humu/5597086 (PMC13741794; doi:10.1155/humu/5597086)
Supplement: Supplementary file 1 [file HUMU-2025-5597086-s001.pdf]

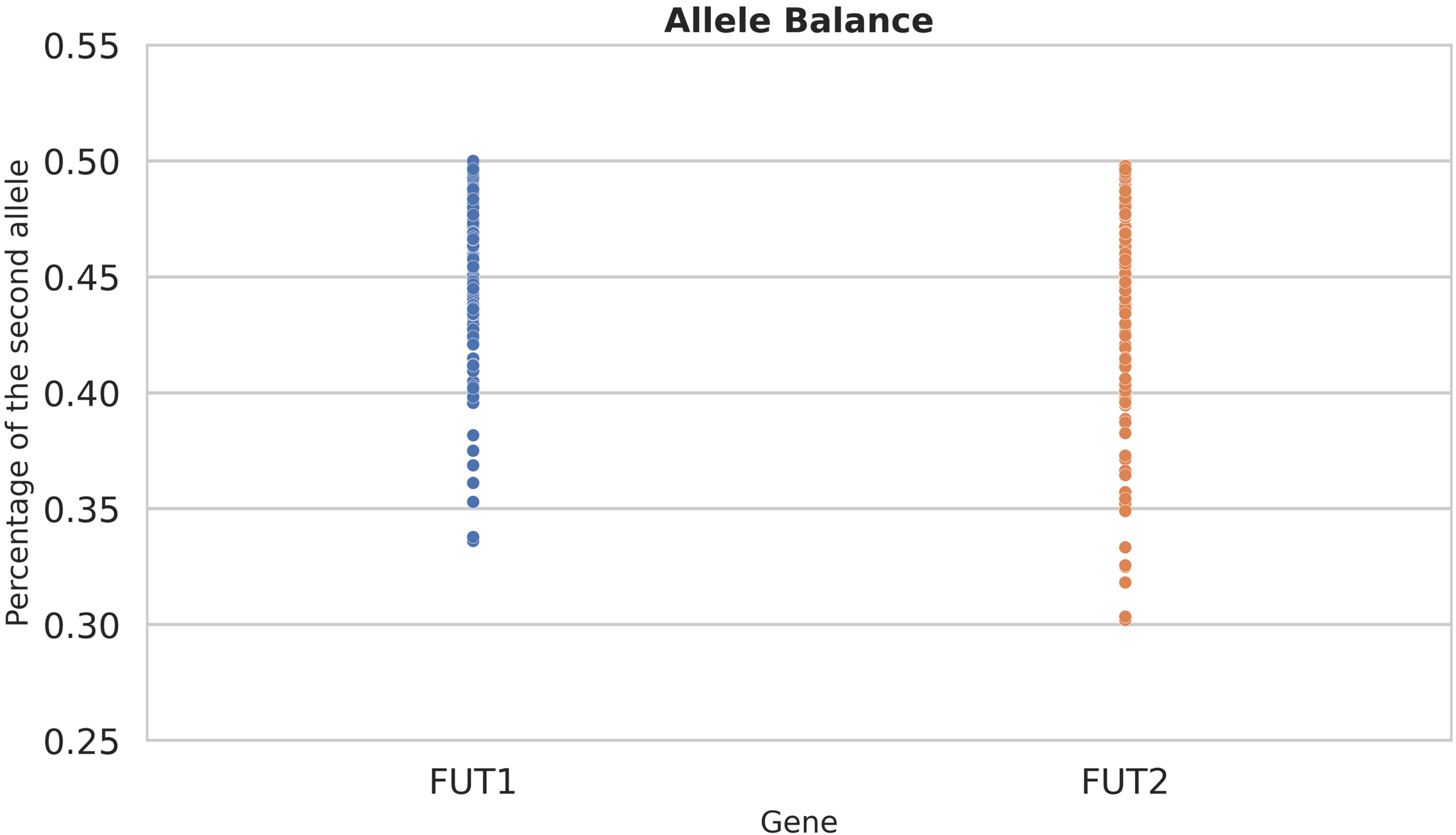
Allele Balance
Percentage of the second allele
0.55
0.50
0.45
0.40
0.35
0.30
0.25
FUT1
FUT2
Gene
